# Biotechnological Interventions for Ginsenosides Production

**DOI:** 10.3390/biom10040538

**Published:** 2020-04-02

**Authors:** Saikat Gantait, Monisha Mitra, Jen-Tsung Chen

**Affiliations:** 1 Crop Research Unit (Genetics and Plant Breeding), Bidhan Chandra Krishi Viswavidyalaya, Mohanpur, Nadia, West Bengal 741252, India; saikatgantait@yahoo.com; 2 Department of Agricultural Biotechnology, Faculty of Agriculture, Bidhan Chandra Krishi Viswavidyalaya, Mohanpur, Nadia, West Bengal 741252, India; monisha25.mitra@gmail.com; 3 Department of Life Sciences, National University of Kaohsiung, Kaohsiung 811, Taiwan

**Keywords:** bioreactor, cell suspension, hairy root, polyploidy, protoplast

## Abstract

Ginsenosides are secondary metabolites that belong to the triterpenoid or saponin group. These occupy a unique place in the pharmaceutical sector, associated with the manufacturing of medicines and dietary supplements. These valuable secondary metabolites are predominantly used for the treatment of nervous and cardiac ailments. The conventional approaches for ginsenoside extraction are time-consuming and not feasible, and thus it has paved the way for the development of various biotechnological approaches, which would ameliorate the production and extraction process. This review delineates the biotechnological tools, such as conventional tissue culture, cell suspension culture, protoplast culture, polyploidy, in vitro mutagenesis, hairy root culture, that have been largely implemented for the enhanced production of ginsenosides. The use of bioreactors to scale up ginsenoside yield is also presented. The main aim of this review is to address the unexplored aspects and limitations of these biotechnological tools, so that a platform for the utilization of novel approaches can be established to further increase the production of ginsenosides in the near future.

## 1. Introduction

**Figure 1:**
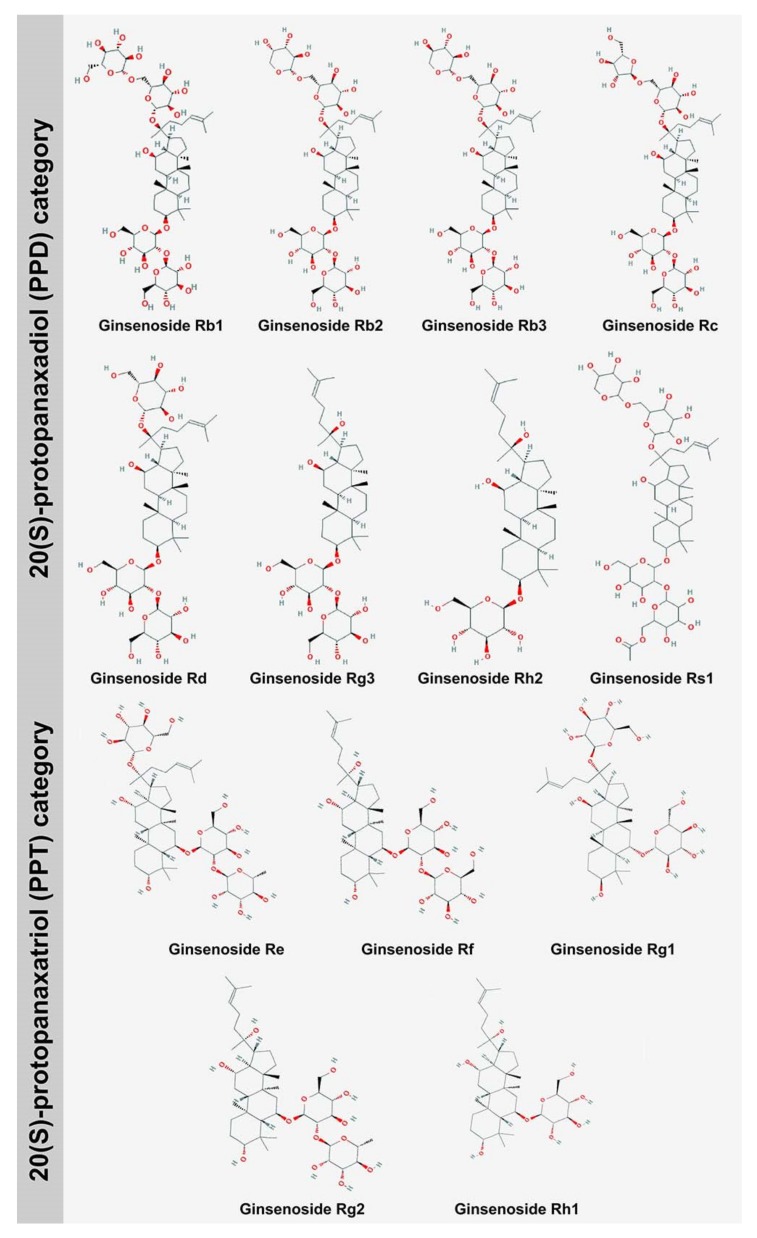
Major structures of ginsenosides belonging to the 20(S)-protopanaxadiol (PPD) and 20(S)-protopanaxatriol (PPT) categories (Structure source: PubChem https://pubchem.ncbi.nlm.nih.gov) (Source: unpublished photograph of Saikat Gantait).

Ginsenosides, commonly called triterpenoids or ginseng saponins, are secondary metabolites bearing immense medicinal importance, especially in the pharmaceutical sector. These secondary metabolites have multifaceted pharmacological properties, owing to their resemblance to steroidal hormones. The amphiphilic nature of ginsenosides allows them to cross the plasma membrane, subsequently inducing signaling cascades that comprise major pathways, e.g., the adenosine-monophosphate–activated protein kinase pathway (which activates B cells). Ginsenosides also trigger receptors, including the glucocorticoid, estrogen, and N-methyl-D-aspartate receptors [1]. Ginsenosides are synthesized mainly by *Panax* species that belong to the family Araliaceae. The plants belonging to the *Panax* genus grow in the Northern hemisphere, and their cultivation is confined to North America. On a commercial basis, ginsenosides have been regarded as profitable drugs that can be utilized for medicinal purposes and have a good stand in the global market. The total revenue achieved in the sales of these metabolites is about 2,000 million US dollars. The major countries where ginsenoside production is commercially exploited are the United States of America, Canada, China, and South Korea. In European countries, ginsenoside production is primarily aimed at manufacturing pharmaceutical drugs, whereas in America, ginsenosides are used to manufacture retail products [2]. The chemical annotation of ginsenoside is ‘Rx’, wherein ‘R’ signifies root, and ‘x’ indicates the chromatographic polarity arranged in alphabetical order. The chemical structure of ginsenosides is common to all the compounds reported till date. The structure consists of 1,2-cyclopentanoperhydrophenanthrene. Ginsenoside compounds can be distinguished from each other on the basis of the number of moieties of sugar attached, the type of sugar, and the linkage position. Ginsenosides are further classified into two categories (Figure 1) based on stereochemistry structure, namely, 20(S)-protopanaxadiol (PPD) (Rb1, Rb2, Rb3, Rc, Rd, Rg3, Rh2, Rs1), wherein an extra carbonyl group is present in PPDs, at the C_6_ position, and 20(S)-protopanaxatriol (PPT) (Re, Rf, Rg1, Rg2, Rh1) [3]. The PPD group of ginsenosides is abundantly found in *Panax quinquefolium*, whereas the PPT group of ginsenosides is commonly found in *Panax ginseng*. There are other types of ginsenoside also available, which are pentacyclic oleane saponin Ro and ocotillol saponin F11. The ocotillol ginsenosides are found mainly in *P. quinquefolium*, whereas the oleane ginsenosides are major constituents of *P*. *ginseng* [4]. Ginsenosides are further divided into two categories, namely, acidic ginsenosides (consisting of four ginsenosides, viz., Rb1, Rb2, Rc, and Rd, which are malonyl derivatives) and neutral ginsenosides, which are actually in fact esterified derivatives. The malonyl ginsenosides are more abundant in *Panax notoginseng* than in *P. ginseng*. The ginsenoside content of a plant is dependent on distinctive factors, like growing conditions, age of the root, root size (which is further dependent on primary roots, secondary roots, and adventitious-root hair). Ginsenoside accumulation is maximum in roots, but new ginsenosides have been isolated from the aerial parts of plant as well, for instance, the floral ginsenosides A–P, derived from the floral buds of *P. ginseng*, and the floral ginsenosides A–E, derived from the floral buds of *P. quinquefolium*. *Gynostemma pentaphyllum* is the only species that does not belong to the Araliaceae family and is a rich source of dammarane triterpene ginsenosides. The conventional approaches of ginsenoside extraction are time-consuming; since the conventional propagation of plants requires approximately six years and is not convenient. Moreover, the conventional method of metabolite extraction requires a long time and the use of methanol, which poses health hazards. The conventional method of extraction involves the basic steps of heating, boiling, and refluxing that can cause the loss of active phytochemicals due to temperature fluctuations and chemical changes induced by reactions such as hydrolysis and oxidation [5]. Thus, to address the shortcomings of the conventional mode of metabolite extraction, cutting-edge biotechnological approaches like tissue culture-mediated mass regeneration technologies, *Agrobacterium*-mediated genetic transformation, and cell suspension culture coupled with elicitation have been implemented in the past three decades to enhance the production efficiency of ginsenosides in a much refined way. These approaches are highlighted in this review.

## 2. Medicinal Uses

Ginsenosides have a broad-spectrum curing capability against several ailments, which is reason enough for them to hold a unique place in the pharmaceutical sector. Ginsenosides possess anti-microbial and anti-fungal properties. They serve as anti-cancerous agents since they restrict metastasis and growth of tumor through a direct cytotoxic action, induce apoptosis, thus preventing tumor invasion, and further restrict chromosome aberrations, which is a prime reason for metastasis [6]. The Rh2 ginsenoside has better anti-cancerous properties than the other ginsenosides. Intravenous application of ginsenoside Rb2 resulted in a decrease of metastasis in the lungs. It possesses immunomodulatory properties that help in the activation of macrophages and lymphocytes, and this provides protection from many infectious diseases. The major ginsenosides with immunomodulatory properties are Rg1, Rg2, Rb1, Re, and Rc. The Rb1 ginsenoside was shown to promote a significant increase of humoral and cell-mediated immunity as a result of the increase of T cells and helper T cells [7]. Anti-inflammatory properties are exhibited by ginsenoside Rg1 in microglial cells of the central nervous system [8]. The anti-inflammatory effects of ginsenosides have even proven to be better than those of the popular drug disodium cromoglycate, which is a commercial anti-allergic drug. Ginsenosides also exhibit anti-allergic properties since they inhibit histamine secretion from mast cells. They also possess membrane-stabilizing properties that restrict membrane disturbances, which is a major mechanism of their anti-allergic properties [9]. Ginsenosides play a major role in the treatment of cardiac ailments by suppressing thrombin production and reducing the activity of sympathetic nerves, thus directly lowering vascular activity and, as a consequence, blood pressure [10]. Ginsenosides release NO that leads to the production of cyclic GMP, which minimizes vascular activity. They also act as regulators of total cholesterol and high-density cholesterol levels, thus preventing chronic diseases like atherosclerosis and other cardiac diseases. The ginsenosides Rg2 and Rg3 are responsible for the inhibition of platelet aggregation via regulating the levels of cyclic GMP and cyclic AMP and suppressing the conversion of fibrinogen to fibrin [11]. They also help in the reduction of hypertension by promoting vasorelaxation. The ginsenosides Rb3 and Rb1 regulate the levels of polyamines that are responsible for cellular growth and regeneration of neural cells [12]. Polyamines are also called stress-based stimuli markers. Rb3 and Rb1 are responsible for blocking the enzyme ornithine carboxylase, which is further responsible for generating polyamines. Ginsenosides play an important role in the treatment of neurological disorders like Alzheimer’s and Parkinson’s diseases. They have also been utilized for the treatment of nervous ailments like amnesia, wherein they enhance cholinergic activity by promoting the uptake of choline, thus improving synaptic transmission [13]. The ginsenoside Rb1 has been shown to increase neural outgrowth, a property that can be utilized for the treatment of dementia. The ginsenosides Rb1 and Rg1 are also responsible for reversing the detrimental effects of cell death and also aid in modulating nerve transmission by further regulating the levels of neurotransmitters [14]. Ginsenosides are also involved in the treatment of several stomach ailments. The ginsenoside Rf has multifold beneficial effects on metabolism, which further contributes to prevent various diseases like lipid disorders, diabetes, and obesity [15].

## 3. Natural Biosynthesis

**Figure 2:**
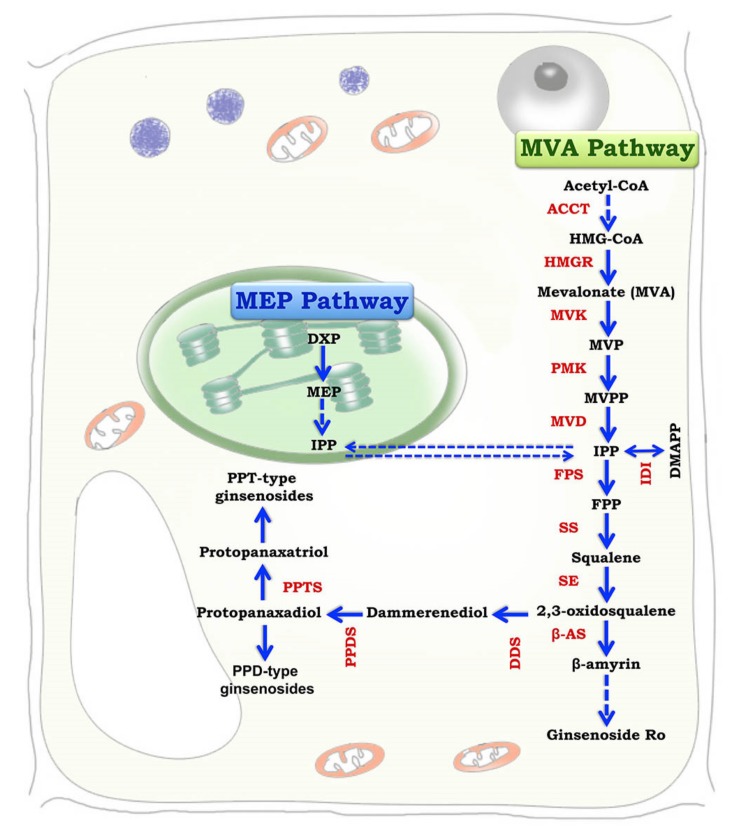
Biosynthesis of ginsenosides via the mevalonic acid (MVA) pathway (inside the cytosol) and the methylerythritol (MEP) pathway (inside plastids) (Concept source: Kim et al. [17]; modified and redrawn by Saikat Gantait).

Ginsenosides are specialized plant metabolites that share precursors with the primary sterol biosynthesis pathway. The biosynthesis of triterpene ginsenosides takes place inside the cytosol and plastids via the mevalonic acid (MVA) pathway and the methylerythritol (MEP) pathway, respectively (Figure 2). Ginsenosides are synthesized via an isoprenoid pathway form the precursors isopentenyl diphosphate (IPP) and dimethyallyl diphosphate (DMAPP) [16,17]. The enzymes responsible for ginsenoside production in biological systems are squalene synthase (SS), farnesyl diphosphate synthase (FPS), and geranyl diphosphate synthase (GPPS). Ginsenosides biosynthesis involves one molecule of DMAPP that binds with two molecules of IPP to form FPP (farnesyl diphosphate), and the combination of two molecules of FPP produces a linear chain of squalene, which contains 30 carbon atoms [18]. The linear molecule squalene is epoxidized to 2,3-oxidosqualene, which is then cyclized to dammarenediol, the specific precursor of ginsenoside in *Panax* spp. It is from this process that ginsenosides can directly be synthesized via oxidation, mediated by cytochrome P450-dependent monooxygenases [19]. Although ginsenoside biosynthesis can occur via both MVA and MEP pathways, ginsenoside production occurs mainly through the MVA pathway, as shown by Schramek et al. [20] through a pulse-chase experiment in a 6-year-old *P. ginseng* plantlet using carbon isotopes. The MVA pathway is a universal pathway active in both eukaryotic and prokaryotic organisms. Inhibition experiments pointed out that the MEP pathway is activated when there is a limited supply of products from the MVA pathway. The MVA pathway is inhibited by light, whereas the MEP pathway is stimulated by light or enhanced by phytochrome signaling. Ginsenosides accumulate in the root epidermis. Histochemical assays showed that ginsenoside accumulation is found in the oil canals of the outer cortex but is totally absent in xylem cells and pith cells. The genes responsible for ginsenoside biosynthesis are expressed mainly in phloem cells, suggesting that these are the major sites for ginsenoside production [3]. Ginsenoside production was also reported to occur in cell organelles like vacuoles, plastids, and peroxisomes in leaves. The ginsenosides thus produced are transported to the root cortex. The transportation of ginsenosides is regulated by a complex cassette transporter, which involves adenosine triphosphate (ATP) [3].

## 4. In Vitro Approaches for Secondary Metabolite Production

### 4.1. Adventitious Shoot Culture

Adventitious shoot culture is a synonymous term for direct organogenesis. Direct organogenesis is defined as the induction of roots and shoots from explants without the formation of an intervening callus. This phenomenon is regulated by the endogenous accumulation of plant growth regulators as well as by their exogenous application [21]. Not many reports on direct organogenesis for ginsenoside estimation are available; the limited few are summarized in Table 1. In addition, there is only a single report on direct organogenesis coupled with ginsenoside estimation in *P. ginseng*, wherein leaves were utilized as explants and inoculated in basal media supplemented with 6-benzyladenine (BA), a cytokinin that promoted the lateral development of shoot buds [22]. Hence, the utilization of a wide array of explants such as shoot tip, nodal segment, hypocotyl, etc., can be employed for the production of ginsenosides in vitro, since this approach is simple and reliable.

### 4.2. Callus Culture, Somatic Embryogenesis, and Regeneration.

Indirect organogenesis or callogenesis is defined as the phenomenon of regeneration of plantlets from a mass of unorganized cells termed callus. Callus is categorized into two groups, namely, friable callus and compact callus, which are employed for suspension culture and regeneration experiments, respectively [21]. There are extensive reports on callus induction and estimation of ginsenosides in the callus cells, which are summarized in Table 2. The utilization of leaves as explants has been adopted owing to their larger surface area. The utilization of auxins in the basal medium leads to the formation of friable calli. The process of regeneration of plantlets from callus is effectuated by the addition of gibberellic acid (GA_3_) and 6-benzylaminopurine (BAP) in the basal medium [23,24]. Compounds like picloram or dicamba can also be used to induce callus from an array of explants, since they possess auxin-mimicking activity.

Somatic embryogenesis is defined as the phenomenon via which somatic embryos are induced from a group of cells that are somatic in origin. The induction of a somatic embryo from callus is regulated by the type and dosage of plant growth regulators used in the growth medium [25]. For ginsenoside production in vitro, there are ample reports available on somatic embryogenesis, which are listed in Table 1. There is also a single instance wherein somatic embryos underwent the process of acclimatization following germination [26].

### 4.3. Cell Suspension Culture

Cell suspension culture is regarded as a convenient approach for the production of secondary metabolites, since it is not season-dependent, and harvesting of cells devoid of biotic contaminants is much easier [37]. Multiple reports on ginsenoside production, based on cell suspension culture in *Panax* spp., are available and are summarized in Table 2. Generally, the induction of callus is initiated with the help of plant growth regulators, mainly auxins, to obtain friable calli [38]. Elicitors are low-molecular-weight compounds that induce secondary metabolite formation in plants by inducing stress-like conditions and have a direct effect on the biosynthetic pathway [39]. Elicitors are also employed for further enhancement of ginsenoside production. Yu et al. [40] used the fungal strain *Alternaria panax*, which acted as a biotic elicitor; the exudates from the fungal cell wall, which contained oligosaccharides along with chitin, aided in the enhancement of ginsenoside accumulation. The utilization of jasmonate compounds also elevated ginsenoside accumulation in the cell cultures, since jasmonates induce oxidative stress in the culture and downregulate many genes, which leads to the augmentation of secondary metabolite levels. Generally, the elicitors aid in stimulating ginsenoside accumulation by activating phenylalanine amino lyase. This enzyme, in turn, helps in the synthesis of defense compounds, which indirectly affects the ginsenoside biosynthetic pathway [41,42,43].

### 4.4. Protoplast Culture

Protoplast culture is regarded as a promising tool for the development of interspecific hybrids of those species that are incompatible when crossed conventionally. In protoplast culture, protoplasts are isolated from the counter parents and are fused to form a hybrid in vitro [21]. There is a sole report available on enhanced ginsenoside production based on protoplast fusion between carrot and American ginseng (*P. quinquefolius*). The hybrid obtained was subjected to high-performance liquid chromatography (HPLC) analysis, and ginsenoside accumulation in the hybrid calli (seven in number) was observed; introgression among these lines enhanced the ginsenoside concentration [49].

### 4.5. Bioreactor: Large-Scale Propagation

Bioreactors are now emerging implements in bioprocessing industries, wherein the optimum environmental conditions are maintained to achieve the required biological products on a large scale. The advantages of bioreactors include a better rate of product multiplication, lesser time for multiplication, and minimum cost [50]. Ginsenoside production in various bioreactors, under different culture conditions, is presented in Table 3. There are various kinds of bioreactors. Stirred-tank bioreactors are the most commonly used (they were utilized by Wang et al. [44] and Kochan et al. [51]), since they allow easier accumulation of cells at various stages due to their large capacities and for their capability to scale up nutrients [52]. The airlift and balloon-type airlift bioreactors are also utilized for ginsenoside production. This type of bioreactors have the additional advantage of better oxygen transfer efficiency with better prediction of flow patterns thus reducing cell shearing [53]. In sprinkle bioreactors, homogeneous culture conditions are usually maintained, and therefore, monitoring becomes much easier [54]. Overall, in all the reports, a pH ranging between 5 to 7 was maintained. Owing to the breakdown of substrate, release of ammonia occurred, which in turn resulted in a decrease of pH; therefore, pH monitoring was a priority [55]. The temperature maintained in the bioreactors was above 20 °C, which is the most favorable temperature for enhanced root biomass and ginsenoside accumulation. Increased aeration rate in bioreactors resulted in an increase in the volume of roots and further metabolite accumulation. The impact of atmospheric gases also determined ginsenoside accumulation, whereby a higher concentration of ethylene and carbon dioxide led to a decrease in ginsenoside production. The increase in ginsenoside accumulation was made possible by the accumulation of nitrate ions and the decrease in ammonia ions. There is a report on the utilization of squalene as an elicitor in bioreactors, wherein squalene resulted in the accumulation of protopanaxatriol groups (the building blocks of ginsenosides) [56].

### 4.6. In Vitro Mutagenesis

Ginsenosides production from in vitro cultures is a popular approach to enhance their production rate. Although somaclonal variants during in vitro cultures are detected at a lower frequency, they are desirable to augment the synthesis of ginsenosides [65]. In vitro mutagenesis incorporates a genotypic change in a culture, and the derived population can be maintained via rigorous subculturing. Cotyledonary explants were inoculated in callus induction medium supplemented with 1 mg/L 2,4-D and 0.1 mg/L kinetin. The induced calli were then exposed to gamma radiations ranging from 10 to 100 Gy (Gray). A dosage of 30 Gy was selected as the adequate dose, and via HPLC analysis, it was confirmed that there was an increase in ginsenoside production in the mutant lines [66]. Similarly, Kim et al. [2] conducted an experiment wherein the in vitro grown calli were exposed to gamma radiations in the 50 Gy range and then cultured in MS media supplemented with 3 mg/L indole butyric acid (IBA). An increase in the concentration of primary ginsenosides in the mutant lines was confirmed subsequently by thin-layer chromatography (TLC) and HPLC analysis. The same was further validated with gene expression studies using RT-PCR, whereby the expression of squalene epoxidase, dammarenediol synthase, and phytosterol synthase genes were enhanced in the mutant lines. There are also reports wherein spontaneous mutation resulted in the overexpression of the *DDS* gene, which is responsible for ginsenoside accumulation. Recently, Le et al. [67] conducted an experiment to determine the sensitivity of mutagens, wherein somatic embryos were exposed to gamma radiation ranging from 20 to 400 Gy, and the optimal radiation dose was standardized at 80 Gy. The gamma-irradiated somatic embryos were germinated in MS medium supplemented with gibberellic acid.

### 4.7. Induction of Polyploidization

The induction of polyploidy or artificial chromosome doubling with the help of anti-mitotic agents is mainly implemented for enhancing the biomass of a plant and, thus consequently amplifying the metabolite profile as well [68]. In *P. ginseng*, in vitro adventitious roots were excised, treated with 100 mg/L colchicine over 60 h and inoculated in MS medium supplemented with 50 mg/L sucrose and 2 mg/L α-naphthalene acetic acid (NAA). After 40 days, the treated roots were subjected to HPLC analysis, whereby the accumulation of ginsenosides was observed in the resultant regenerated octaploid plantlets, suggesting that chromosome doubling can enhance biomass and ginsenoside accumulation, simultaneously [69]. Hence, based on these studies, it is evident that polyploidization can be a viable approach to increase ginsenosides yield. The utilization of anti-mitotic agents like oryzalin or trifluralin can ensure a successful polyploidization. In addition, flow cytometry analysis can also be used to confirm the polyploidy level in anti-mitotic agent-treated explants, in addition to the conventional chromosome counting method.

### 4.8. Hairy Root Culture

Genetic transformation with the help of *Agrobacterium rhizogenes* gives rise to transformed hairy roots. The induced hairy roots often exhibit a comparable or higher biosynthetic capacity for secondary metabolite production with respect to non-transformed roots, owing to the presence of auxin-responsive genes and overexpression of *rol* genes that can further lead to an increase in biomass [70]. This observation gave rise to the development of a new direction associated with the use of hairy roots for the production of these secondary metabolites. Hairy root culture has innumerable advantages. For instance, the growth phase of the culture remains stable throughout, and the culture possesses high genetic stability and negative geotropism. Even under control conditions, hairy roots grow at a higher rate than normal adventitious roots. The most positive aspect of hairy roots is that they exhibit a higher biosynthetic rate than the mother plant [71]. There are extensive reports on ginsenoside production using this technique, some of which are listed in Table 4. The leaf is the explant of choice in most cases, since it possesses a large surface area and allows a more effective adhesion of the bacterial suspension, resulting in better chances of transformation [72]. As for the maintenance medium, MS is prevalently employed due to the presence of a high amount of ammonia and nitrate ions [73]. Cefotaxime is recurrently used in all the experiments for genetic transformation due to its broad-spectrum activities against Gram-positive and Gram-negative bacteria [74]. Molecular confirmation of gene integration was carried out via PCR amplification of *rol* genes, which are responsible for the positive regulation of metabolite production [54,75]. Kim et al. [66] performed transcriptional profiling of putative genes for ginsenosides production, viz., *PgSS* (squalene synthase), *PgSE* (squalene epoxidase), and *PNA* (dammarenediol synthase-II) genes. In genetically transformed hairy roots, the presence of ocotillol ginsenosides was detected at a considerable concentration when compared to the roots collected from conventionally grown ex vitro plants.

## 5. Conclusions and Future Prospect

**Figure 3:**
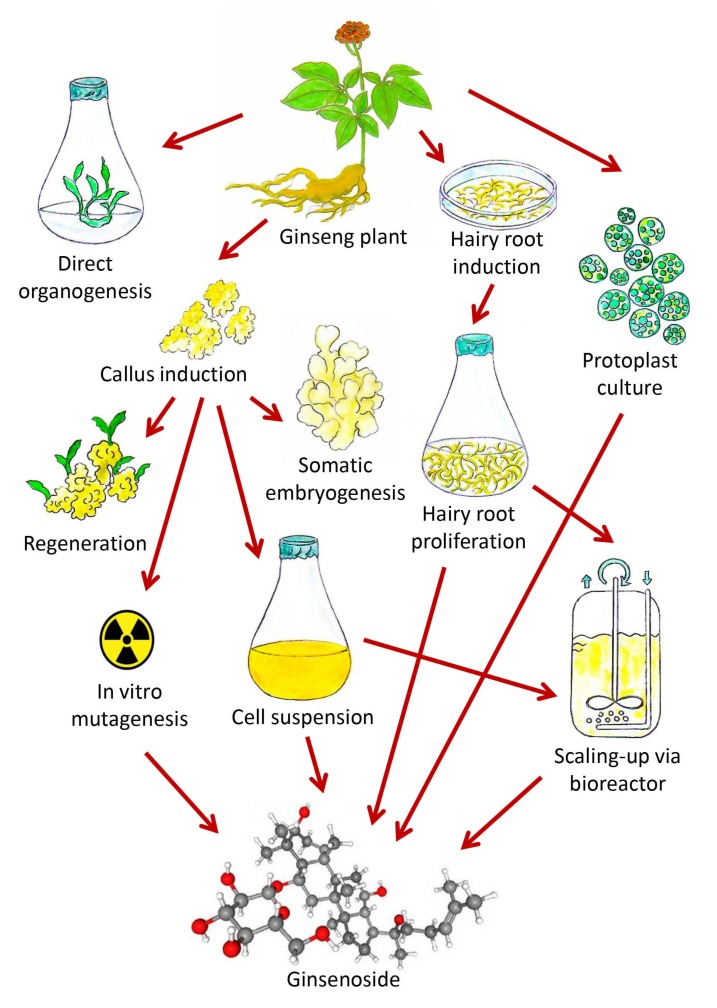
Diagram showing the enhanced production of ginsenosides through various in vitro biotechnological approaches (Source: unpublished photograph of Saikat Gantait).

The most recent biotechnological advances regarding ginsenoside production under in vitro conditions have been highlighted (Figure 3) and described extensively in this review. There are only a handful of reports available on direct and indirect organogenesis experiments, wherein tissue culture-mediated technologies like direct organogenesis, indirect organogenesis, and somatic embryogenesis have not been extensively investigated for the purpose of ginsenoside production, and the quantitative estimation of ginsenosides via HPLC or high-performance thin-layer chromatography (HPTLC) has not been attempted as well. The utilization of additives, elicitors, or precursors in organogenesis experiments and somatic embryogenesis experiments needs to be addressed properly, since these are compounds that can interfere with the signaling pathways that directly or indirectly affect ginsenoside biosynthesis and can enhance their production. There are ample reports on cell suspension cultures and bioreactors, yet the use of elicitors needs to be explored more extensively, since these compounds may greatly contribute to ginsenoside amelioration. These methods have been mainly implemented to increase plant biomass and to further promote metabolite production in a much shorter period of time and are not season-dependent. There are several reports on hairy root culture and the use of other approaches of genetic transformation like direct methods that employ gene guns, particle bombardment, etc., or the use of *Agrobacterium tumifaciens*. On the other hand, few reports on polyploidy that are available till date and this technique needs to be studied furthermore with the extensive applications of antimitotic agents like oryzalin or trifluralin in variable concentrations at different exposure times to enhance the production of ginsenosides. In vitro mutagenesis is the most innovative approach that has gained recognition only recently and needs to be analyzed furthermore to explore its beneficial effects for the increased production of ginsenosides. In conclusion, this review provides an overview of the current biotechnological advancements for ginsenoside production in vitro and also highlights the main unexplored research areas that need to be addressed in the near future.

## Figures and Tables

**Table 1:** Factors involved in and their influence on ginsenoside production during indirect organogenesis.

*Panax* sp.	Explant	Surface Sterilization	Basal Medium	Carbon Source	PGRs (mg/L or μM*)	Other Media Additives (mg/L or g/L*)	Culture Condition [Temp; PP; LI lux or PPFD^#^; RH]	Response	Acclimatization [Substrate Used (v/v); % Survival]	Ginsenoside Yield	Reference
*P. ginseng*	Leaf	70% EtOH 30 sec → 0.625% NaOCl 1 min	MS	3% sucrose	10* NAA + 9* 2,4-D	NM	NM; 12 h; 24^#^; NM	Somatic embryo	NM	NM	[23]
					3* GA3 + 5* BAP			Shoot regeneration and rooting			
*P. ginseng*	Anther	70% EtOH 30s → 2% NaOCl 1 min with drops of Tween 20	MS	9% sucrose	4.53* 2,4-D	NM	25 ± 1 °C; NM; NM; NM	Callus induction	NM	NM	[24]
					28.9* GA3			Shoot regeneration			
*P. pseudoginseng*	Rhizome	70% EtOH 30s → 2% NaOCl 1 min with drops of Tween 20 → 0.1% HgCl_2_	MS	3% sucrose	2.5 2,4-D + 2.5 BAP	NM	NM	Somatic embryo	Black garden soil + compost+ leaf litter (1:1:1); 70%	NM	[26]
					1 GA3			Somatic embryo generation			
*P. ginseng*	Cotyledon	70% EtOH → 1% NaOCl	MS	3% sucrose	NM	40 mM NH_4_NO_3_	22 ± 2 °C; 16 h; 24^#^; NM	Callus induction	NM	4.39 mg/g	[27]
*P. quinquefolium*	Root	NM	MS	3% sucrose	2.5 2,4-D	NM	NM	Callus induction	NM	NM	[28]
*P. ginseng*	Root	70% EtOH 30s → 1% NaOCl 1 min	SH	3% sucrose	1 2,4-D + 0.1 KIN	NM	25 ± 2 °C; NM; NM; NM	Callus induction	NM	83.37 mg/L	[29]
*P. ginseng*	Root	70% EtOH 30 sec → 20% NaOCl 1 min with drops of Tween 20	MS	3% sucrose	2 2,4-D	0.1%(w/v) myo inositol	25 °C; NM; NM; NM	Callus induction	NM	NM	[30]
*P. ginseng*	Root	75% EtOH	MS	3% sucrose	2 2,4-D + 0.5 KIN	NM	23 ± 2 °C; NM; NM; NM	Callus induction	NM	132.9 mg/L	[31]
*P. ginseng*	Root	NM	MS	5% sucrose	25* IBA	NM	22 ± 1 °C; NM; NM; NM	Callus induction	NM	7.3 mg/L	[32]
*P. ginseng*	Leaf	70% EtOH 30 sec → 0.1% HgCl_2_ for 1 min	MS	3% sucrose	4 BAP	NM	25 ± 2 °C; 16 h; 24^#^; 80	Shoot induction	NM	NM	[33]
*P. quinquefolium*	Root	70% EtOH 30 sec	MS	3% sucrose	1 2,4-D + 0.25 KIN	NM	23 ± 2 °C; NM; NM; NM	Callus induction	NM	NM	[34]

Abbreviations: 2,4-D, 2,4-dichlorophenoxyacetic acid; BAP, 6-benzylaminopurine; MS, Murashige and Skoog [35]; NAA, α-naphthalene acetic acid; IAA, indole acetic acid; KIN, kinetin; LI, light intensity; RH, relative humidity; PP, photoperiod; IBA, indole butyric acid; NA, not applicable; NM, not mentioned, PGR, plant growth regulator; PP, photoperiod; SH Schenk and Hildebrandt [36].

**Table 2:** Factors involved in and their influence on ginsenoside production in cell suspension culture.

*Panax* sp.	Basal Media	Carbon Source	PGR (mg/L or *μM)	Elicitor (μM/*mg/L)	Culture Conditions (Temp, PP, RH, LI, rpm)	Yield	References
*P. ginseng*	MS	3% sucrose	0.1 KIN + 1 2,4-D	NM	25 °C, dark, NM, NA, NM	54 mg/g	[30]
*P. ginseng*	MS	4% sucrose	1 2,4-D	NM	24 ± 1 °C, dark, NM, NA, NM	3.08 mg/g	[38]
*P. ginseng*	MS	3% sucrose	0.25 KIN	6* *Alternaria panax*	25 °C, dark, NM, NA, NM (30 days)	276 mg/L	[40]
*P. ginseng*	MS (no NH_4_NO_3_)	5% sucrose	2 NAA	150 MJ	22 °C, dark, NM, NA, 110 rpm (40 days)	48 mg/g	[41]
*P. notoginseng*	MS	NM	NM	100 2-hydroxyethyl jasmonate	NM, NM, NM, NM, NM	32.7 mg/L	[42]
*P. ginseng*	MS	3% sucrose	2 IBA + 0.1 KIN	2* JA	NM, dark, NM, NA, 100 rpm	255 mg/L	[43]
*P. quinquefolium*	MS	3% sucrose	0.25 KIN + 1 2,4-D	NM	23 ± 2 °C, dark, NA, 120 rpm (90 days)	3.36 mg/g	[44]
*P. vietnamensis*	MS	3% sucrose	0.1 KIN + 3 2,4-D	NM	25 °C, dark, NM, NA, 105 rpm	5.7 mg/g	[45]
*P. ginseng*	MS	3% sucrose	25* IBA	NM	25 °C, dark, NM, NA, 100 rpm	5.4 mg/g	[46]
*P. ginseng*	MS	3% sucrose	0.5 BAP + 2 2,4-D	500* CH	25 °C, dark, NM, NA, 100 rpm	NM	[47]
*P. quinquefolium*	MS	3% sucrose	0.002 TDZ + 0.2 2,4-D	NM	26 ± 2 °C, 90%, NM, 40 μE/m^2^/s, 100 rpm (40 days)	29.11 mg/g	[48]

Abbreviations: MJ, methyl jasmonate; JA, jasmonic acid; CH, casein hydrolysate; NM, not mentioned; NA, not applicable; TDZ, thidiazuron.

**Table 3:** Factors involved in and their influence on ginsenoside production during regeneration via bioreactors.

Species	Type of Bioreactor	Basal Media	PGR (mg/L)	Elicitor/Additives	Culture Condition (Temp, PP, Other)	Ginsenoside Yield	References
*P.quinquefolius*	Balloon type airlift	MS	5 IBA	4 mg/L *Alternaria panax*	26 °C, dark, 100 vvm	276 mg/L	[40]
*P.quinquefolium*	Stirred tank	MS	0.25 KIN + 1 2,4-D	100 mg/L Lactoalbumin hydrolysate	23 ± 2 °C, 1 vvm	31.52 mg/L	[44]
*P.quinquefolium*	Stirred tank	MS	0.1 KIN + 1 2,4-D	NM	26 °C, 100 vvm	9 mg/g	[51]
*P.quinquefolium*	Nutrient sprinkle	B5	NM	NM	26 ± 2 °C	32.25 mg/g	[54]
*P. ginseng*	Airlift	MS	NM	10 mM Copper sulphate	0.1 vvm, 23 ± 1 °C	12.42 mg/g	[56]
*P. notoginseng*	Airlift	MS	NM	1 mM copper, 3.75 mM phosphate	Aeration rate: 0.8 vvm	1.75 g/L	[57]
*P. ginseng*	NM	MS	NM	18.5 mH NO_3_^-^	NM	9.9 mg/g	[58]
*P. ginseng*	Balloon type bubble	MS	7 IBA + 0.5 KIN	200 μM MJ	25 °C, dark	8.82 mg/g	[59]
*P. ginseng*	Balloon type airlift	MS	7 IBA + 0.5 KIN	20 ppm Ethylene	NM	NM	[60]
*P. ginseng*	Balloon type airlift	MS	5 IBA + 0.5 KIN	200 μM MJ and salicylic acid	NM	NM	[61]
*P. ginseng*	NM	MS	24.6 μM IBA	NM	0.1 vvm	1.91 mg/g	[62]
*P.quinquefolium*	Nutrient sprinkle	B5	NM	NM	26 °C, dark	12.45 mg/g	[63]
*P.quinquefolium*	Nutrient sprinkle	B5	NM	250 μM MJ	26 °C	24.77 mg/g	[64]

**Table 4:** Factors involved in and their influence on ginsenoside production during hairy root culture.

Species	Explant	Strain	Basal Media (for induction)	Antibiotics (mg/L)	Elicitors (mg/L)	Basal Media (for maintenance)	Ginsenoside Yield	Reference
*P. ginseng*	Root	A4	NM	NM	NM	MS + 3% sucrose	3.62 mg/g	[76]
*P. ginseng*	Root	A4	NM	NM	MJ	SH	NM	[77]
*P. ginseng*	Cotyledon	R1000	NM	800 cefotaxime	NM	½ MS	NM	[78]
*P. ginseng*	Rhizome	A4	YEB	500 cefotaxime	NM	SH	72.9 mg/L	[79]
*P. ginseng*	NM	A4	NM	NM	NM	NM	17.12 mg/g	[80]
*P. ginseng*	Root	KCTC 2703	Nutrient broth	300 cefotaxime	2 JA	½ MS	2 mg/L	[81]
*P. ginseng*	Root	NM	NM	NM	0.1 mM MJ	½ MS	6.83 mg/g	[82]
*P.quinquefolium*	Leaf	ATCC 15834	NM	NM	NM	B5	3 mg/g	[83]
*P.quinquefolium*	Leaf	ATCC 15834	NM	500 ampicillin	NM	B5	9 mg/g	[84]
*P. vietnamensis*	Shoot tip	ATCC 15834	NM	250 cefotaxime	NM	½ SH	10 g/L	[85]

Abbreviations: B5, Gamborg et al. [86].
